# Joint Working of the Two Corporations

**Published:** 1919-10-11

**Authors:** 


### BRITISH RED CROSS AND THE ORDER OF ST. JOHN.

### Joint Working of the Two Corporations.

Under the agreement, which was entered into on September 4, a Joint Council is set up to control generally the work of the British Red Cross Society and the Order of St. John. This Joint Council will consist of the Grand Prior of the Order of St. John and the Chairman of the Council of the Red Cross Society, and not more than forty-eight other members.

THE NEW COUNCIL'S CONSTITUTION.

The first Joint Council will be constituted as follows:

(*a*) Twenty-four members to be appointed by the Order, of whom the first will be the following: Adeline Duchess of Bedford, Ethel Lucy, Lady Perrott, R.R.C., Colonel Sir Herbert Jekyll, K.C.M.G., Colonel Sir Herbert Charles Perrott, Bt., C.H., C.B. the Earl of Plymouth, G.B.E., C.B., Lieut.-Colonel Sir Richard Carnac Temple, Bt., C.B., C.I.E., Sir John Prescott Hewett, G.C.S.I., K.B.E., C.I.E., the Earl of Ranfurly, G.C.M.G., the Hon. Sir William Henry Goschen, K.B.E., the Right Hon. Evelyn Cecil, P.C., M.P., Sir William Henry Bennett, K.C.V.O., F.R.C.S., Sir Mackenzie Dalzell Chalmers, K.C.B., C.S.I.; together with such other twelve persons as the Order shall nominate.

(*b*) Twenty-four members to be appointed by the Society of whom the first will be the following: Her Royal Highness the Princess Christian, G.B.E., the Marchioness of Lansdowne, C.I., C.H., Georgina Countess of Dudley, R.R.C., Viscountess Northcliffe, Sir Ernest Clarke, Sir William Edmund Garstin, G.C.M.G., G.B.E., Sir Robert Arundell Hudson, G.B.E., Sir Walter Roper Lawrence, Bt., G.C.I.E., G.C.V.O., Mr. Edward A. Ridsdale (Vice-Chairman of the Executive Committee), the Hon. Sir Charles Russell, Bt., the Hon. Sir Arthur Stanley, G.B.E., C.B., M.V.O. (Chairman of the Executive Committee), Sir Frederick Treves, Bt., G.C.V.O., C.B., etc. (Vice-Chairman of the Council); together with such other twelve persons as the Society shall nominate.

At a meeting in May 1920, and at a meeting in May in every subsequent year, one-third of the members of the Joint Council appointed by the Order and one-third of the members of the Joint Council appointed by the Society will retire, but will be eligible for re-election. The Order and the Society respectively will decide as to which of their respective appointees are due for retirement, and will before the next ensuing Joint Council meeting nominate their successors. Any casual vacancy caused by the death or resignation during his or her period of office of any member of the Joint Council will be filled on the nomination of the body to which the member so dying or resigning belonged.

OBJECTS.

The objects of the Joint Council will be as follows:

(*a*) To control and direct the work and objects (whether within or beyond the United Kingdom) of the Order and Society respectively, and by a regulation of activities, whether through devolution to the Order or Society or otherwise, to prevent overlapping, duplication or waste of energy, and ensure harmonious and efficient co-operation between the Order and the Society in carrying out their respective objects for the time being.

(*b*) To control (subject to the authority of the Finance Committee) the expenditure and management of such funds as it may be entrusted with.

(*c*) To promote so far as practicable within the means in its power the improvement of health, the prevention of disease, and the mitigation of suffering throughout the world, whether in peace or war, but so that no appeal for funds or subscriptions will be issued by the Order or the Society without the sanction of the Joint Council or of any authority in any particular area to which it may from time to time delegate the power.

INDIVIDUALITY PRESERVED.

The control and direction of the work and objects of the Order and the Society are to be carried out in such manner as to preserve the individuality of the Order and the Society, and so as not to interfere with their corporate powers and limitations, or with the activities of any now existing oversea organisation of either body without the approval of such organisation.

POWERS IN GENERAL MEETING.

The Joint Council in general meeting will have the following powers:

(*a*) To appoint from among its own members a Chairman and a Vice-Chairman, who will both hold office for one year, but will be eligible for re-election, and will *ex officio* be members of the Committee of Management, the Finance Committee, and all Sub-Committee, but so that both offices shall not be held by representatives of the same body at the same time.

(*b*) To make such rules and regulations for its own procedure as it shall think fit.

(*c*) To decide on the general policy to be adopted with a view to carrying out the objects for which the Joint Council was established.

(*d*) To delegate any of its powers to the Committee of Management, the Finance Committee, or any Sub-Committee, or to any organisation for a county or group of counties.

(*e*) To establish Joint Committees throughout England, Scotland, Ireland, Wales, or elsewhere, for the development of its work.

(*f*) To elect from the members of the Joint Council and similarly at the meeting in May in each year, a Committee of Management with such powers and duties as shall from time to time be delegated to it by the Joint Council.

(*g*) To appoint annually a Finance Committee, to whom all questions of finance shall be referred.

(*h*) To fill any casual vacancy occurring through the death or resignation of a member of the Committee of Management or of the Finance Committee during its period of office by the appointment of any member of the Joint Council belonging to the same body as the member of the Committee of Management or of the Finance Committee so dying or resigning.

(*j*) To appoint (if invited) representatives to any international Committees which may be formed for the furtherance of any of the objects of the Order or the Society.

COMMITTEES.

The Committee of Management will be selected from the Joint Council, and will consist of eight members of the Order and eight members of the Society, who will hold office for one year, but will be eligible for re-election, except that the members elected in 1919 will hold office until the meeting in May 1920.

The Finance Committee will consist of not more than eight members (exclusive of *ex officio members*), four of whom will be selected from the members of the Joint Council appointed by the Order and four from the members of the Joint Council appointed by the Society. The first Finance Committee will hold office until the meeting of the Joint Council in May 1920, but will be eligible for re-appointment. No liabilities are to be incurred or payments made by the Joint Council or the Committee of Management except with the approval of the Finance Committee.

